# Lateral Flow Immunoassay to Detect the Addition of Beef, Pork, Lamb, and Horse Muscles in Raw Meat Mixtures and Finished Meat Products

**DOI:** 10.3390/foods9111662

**Published:** 2020-11-13

**Authors:** Elena A. Zvereva, Demid S. Popravko, Olga D. Hendrickson, Natalia L. Vostrikova, Irina M. Chernukha, Boris B. Dzantiev, Anatoly V. Zherdev

**Affiliations:** 1 A.N. Bach Institute of Biochemistry, Research Center of Biotechnology of the Russian Academy of Sciences, Leninsky prospect 33, 119071 Moscow, Russia; zverevaea@yandex.ru (E.A.Z.); dspopravko@mitht.ru (D.S.P.); odhendrick@gmail.com (O.D.H.); dzantiev@inbi.ras.ru (B.B.D.); 2 Gorbatov Federal Research Center for Food Systems of the Russian Academy of Sciences, Talalikhina Street 26, 109316 Moscow, Russia; n.vostrikova@fncps.ru (N.L.V.); imcher@inbox.ru (I.M.C.)

**Keywords:** skeletal troponin I, lateral flow assay, colloidal gold, mammalian meat, quality control

## Abstract

A lateral flow immunoassay for sensitive detection of skeletal troponin I (TnI) as a specific, thermostable marker of muscle tissue was developed. Due to the antibodies’ choice, the assay specifically detects mammalian TnI (in beef, pork, lamb, and horse) but does not detect bird TnI (in chicken or turkey), thus enabling differentiation of these types of raw meat materials. The assay is based on a sandwich format of the analysis using gold nanoparticles as labels. The time of the assay is 15 min, and the TnI detection limit is 25 ng/mL. A buffer solution is proposed for efficient extraction of TnI from muscle tissues and from finished meat products that have undergone technological processing (smoking–cooking–smoking, cooking and smoking). The possibility of detecting beef addition in minced chicken down to 1% was demonstrated.

## 1. Introduction

In recent decades, the rapid control of the composition of food products and identification of undeclared components has been an acute issue. Consumers worldwide are concerned about mass falsifications of food products, in particular meat products, as a result of numerous reports highlighting potential risks to human health and serious financial losses [1,2]. Falsifications of meat foodstuffs not only change the properties of finished products, but also can violate consumer preferences or dietary restrictions and be dangerous to people’s health and well-being (e.g., halal products, allergenic compounds). In addition, falsified composition, for example by adding beef or pork to a vegetarian product or to a poultry meat product recipe, may lead to contamination of finished products with pathogens specific to mammalian meat [3].

Sensitive, rapid and productive analytical methods are necessary to control the composition of meat products. Microstructural or histological methods are not effective for the analysis of finished meat products that have gone through numerous stages of technological processes. Molecular genetic methods, electrophoresis, and various types of chromatography cannot be used as screening methods because they are laborious and time-intensive and their implementation requires special laboratories, sophisticated equipment and highly skilled personnel [4].

Immunoassays, primarily the enzyme-linked immunosorbent assay (ELISA) and lateral flow immunoassay (LFIA), are effective tools for characterizing the composition of food products. ELISA is a highly sensitive and specific method that allows for quantitative characterization of the target compound but is a lengthy technique [5,6]. Neogen (Lansing, MI, USA), ELISA Technologies (Gainesville, FL, USA), Microbiologique (Seattle, WA, USA), and other companies have developed ELISA tests specific to certain types of meat. For example, tests provided by Microbiologique detect pork [7], horse [8], beef [9], and poultry [10] meat in meat of other species with a sensitivity of 0.1%. The minimum analysis time is 70 min.

LFIAs allow quick, direct content identification and the evaluation of different types of raw materials or contaminants without additional equipment, both in industrial and home conditions. Because the necessary analytic reagents have been pre-applied to the test strip, their contact with the sample is sufficient for all LFIA processes, including final detection of colored zones [11]. Current applications of LFIAs for meat products are limited to controlling veterinary drugs and identifying prohibited components (for example, in halal foods) [12,13]. To identify meat sources, proteins or peptides are often used as markers—for example, myoglobin [14], troponin I [15], type 1 myosin light chains, muscle carbonic anhydrase, and muscle enolase [16]. The biomarkers selected for commercial lateral flow tests are not specified as the know-how of manufacturers. Moreover, the available commercial tests are focused on the identification of specific animals, whereas tools to distinguish different kinds of sources (such as low-cost bird meat and high-cost animal meat) are not presented.

The aim of the study was to develop an LFIA for assessing the content of mammalian muscle tissue in meat products and comparing the obtained values with the declared composition of the products. Troponin I skeletal isoform (TnI) was chosen as a detectable biomarker due to its specificity for muscle tissue and thermal stability [15]. This research is a continuation of our previous development of TnI ELISA for controlling the composition of meat foodstuffs [17].

## 2. Materials and Methods

### 2.1. Chemicals

Bovine skeletal troponin I and monoclonal antibodies against TnI clones IS7 and 6F9 were purchased from HyTest (Turku, Finland, hytest.fi). Rabbit anti-mouse immunoglobulins were obtained from Imtek (Moscow, Russia). Peroxidase-labeled antibodies against mouse immunoglobulins G were obtained from Jackson ImmunoResearch (Cambridgeshire, UK). Sodium citrate, Tween-20, Triton X-100, 3,3′,5,5′-tetramethylbenzidine (TMB), sucrose, and sodium azide were obtained from Sigma Chemicals (St. Louis, MO, USA). All other salts, solvents, and other chemicals of analytical grade came from Khimmed (Moscow, Russia). ELISAs were conducted on Costar 9018 96-well polystyrene microplates (Corning, NY, USA).

### 2.2. Biotinylation of Antibodies

Covalent labeling of the IS7 anti-TnI monoclonal antibody with biotin was performed as described in [18]. A total of 4.8 μL of biotin N-hydroxysuccinic ester (3.1 mg/mL) was mixed with 0.21 mL of antibody IS7 (3.13 mg/mL) and incubated at room temperature for 2 h, followed by dialysis against 50 mM K-phosphate buffer, pH 7.4, with 0.1 M NaCl (PBS). The biotinylated antibodies were separated from low-molecular-weight compounds using Amicon Ultra Centrifugal Filters 10 K (Merck Millipore, Burlington, MA, USA) by washing with PBS three times with centrifugation at 10,000× *g* (15 min, room temperature).

### 2.3. Sandwich ELISA

The sandwich ELISA was implemented as described in [17]. The analysis included the interaction of the sample with antibody 6F9 immobilized on the surface of the microplate (2 μg/mL in PBS) and with biotinylated antibody IS7 (2 μg/mL) used for detection. These stages, each lasting 1 h, were performed in PBS containing 0.05% Triton X-100 (PBST) and 0.5 M KCl. All stages were accompanied by the four washes with PBST. After that, streptavidin–horseradish peroxidase conjugate, diluted 1:5000, was introduced into the system, followed by incubation for 1 h and determination of the activity of the peroxidase label associated with the carrier. For this purpose, the substrate (0.1 M Na-citrate buffer, pH 4.0, with 1.8 mM H_2_O_2_ and 0.42 mM TMB) was incubated for 15 min, and 1 M H_2_SO_4_ was added. The final optical density at 450 nm was measured by a Zenyth 3100 reader (Anthos Labtec Instruments, Wals, Austria).

### 2.4. Preparation of Gold Nanoparticles and Their Characterization

Gold nanoparticles (GNPs) with an average diameter of 40 nm were synthesized by reducing chloroauric acid using sodium citrate, as described in [19].

The obtained GNPs were dropped onto Formvar film-coated grids (300 mesh) and analyzed using transmission electron microscopy on a JEM-100C microscope (Jeol, Tokyo, Japan). Microphotographs were obtained at a voltage of 80 kV and 66,000–100,000 zoom. The Image Tool 3.0 program (University of Texas Health Science Center, San Antonio, TX, USA) was used to analyze the digital images.

### 2.5. Immobilization of the Antibodies on the GNPs

The antibody–GNP conjugates were prepared, as described in [18]. A concentration of 5 μg/mL was selected for conjugation of the IS7 and 6F9 antibodies.

### 2.6. Production of Test Strips

The following membranes were characterized as potential compounds of test strips: CNPC-SS12, GFB-R4, PT-R7 and AP045 (Advanced Microdevices, Ambala Cantt, India), Millipore GFDx203000, HF090, and HF120 (Merck, Darmstadt, Germany), and membrane 8951 (Ahlstrom-Munksjö, Helsinki, Finland). Finally, a CNPC-SS12 working membrane with a 15 µm pore size, a GFB-R4 sample membrane, an AP045 adsorption membrane, and a fiberglass membrane 8951 were selected to manufacture the test strips.

Reagents were immobilized on the membranes at a rate of 0.1 µL per mm using an Iso-Flow automated dispenser (Imagene Technology, Hanover, NH, USA). The load of the conjugate of GNPs and antibodies was 27 µL for 1 cm of glass fiber membrane in 10 mM Tris buffer, pH 8.5, with 1% BSA, 1% sucrose, 0.05% Tween-20, and 0.05% sodium azide. After dispensing, all membranes were dried for at least 20 h and then fixed on a plastic pad.

The test zone was formed using antibody IS7 or 6F9 (2.5 mg/mL diluted in 10 mM Tris-HCl buffer, pH 9.0), and the control zone was formed using rabbit anti-mouse immunoglobulins (0.5 mg/mL in PBS). At a dilution corresponding to *D*_520_ = 3.0, the conjugate of GNPs and antibodies was immobilized onto a glass fiber membrane.

### 2.7. Sample Preparation for the Immunoassay

The raw meat, including beef, pork, lamb, horse, and poultry (chicken, turkey), was purchased in supermarkets. Beef- and pork-based cooked sausages with confirmed composition were produced at the stand for the manufacturing of meat products of the Gorbatov Federal Research Center for Food Systems of the Russian Academy of Sciences (Tables S1 and S2). Vegetarian sausages produced by Russian manufacturers and purchased in Moscow supermarkets were used as reference products. To obtain poultry samples spiked by mammalian meat, ground beef was mixed with ground chicken (20%/80%, 5%/95%, 2%/98%, 1%/99%, 0.5%/99.5% *g*/*g*).

The prepared sausages differed in their composition and casing. Before grinding, the meat was frozen (−2 °C), the meat was ground and finely ground, then the sausages were molded and knitted. Heat treatment included the stages of roasting (90 ± 10 °C, 90 min), boiling (80 ± 5 °C, 80 min), and smoking (43 ± 7 °C, 20 min). The sausages were dried for 36 h in drying chambers at 10–12 °C and a relative humidity of 76.5 ± 1.5%.

The extraction was carried out as described in [17]. One hundred milligrams of homogenized meat (raw meat or meat product) were mixed vigorously (vortex) with 2 mL of PBS, 0.1% Triton X-100, and 0.5 M KCl for 10 min. The mixture was centrifuged for 5 min at 5000× *g* at room temperature. The obtained supernatants were incubated at 100 °C for 3 min, centrifuged for 5 min at 7000× *g*, and used for the TnI LFIA.

### 2.8. Performance of the LFIA

Test strips were immersed into the solutions and incubated for 15 min prior to detection. All the measurements were performed in triplicate for statistical processing. The strips were then scanned using a flatbed scanner (Canon Lide 90) with a 600 dpi resolution. TotalLab software (TotalLab, Newcastle upon Tyne, UK) was used to process the test strip images. Linear approximations of the test line’s color intensity dependence on the TnI concentration were constructed. The cutoff value (limit of detection, LOD) of the LFIA was interpreted as the minimum concentration of bovine TnI causing a reliable coloration in the test zone.

## 3. Results and Discussion

### 3.1. Obtaining and Characterization of Specific Reagents for the LFIA

**Figure 1:**
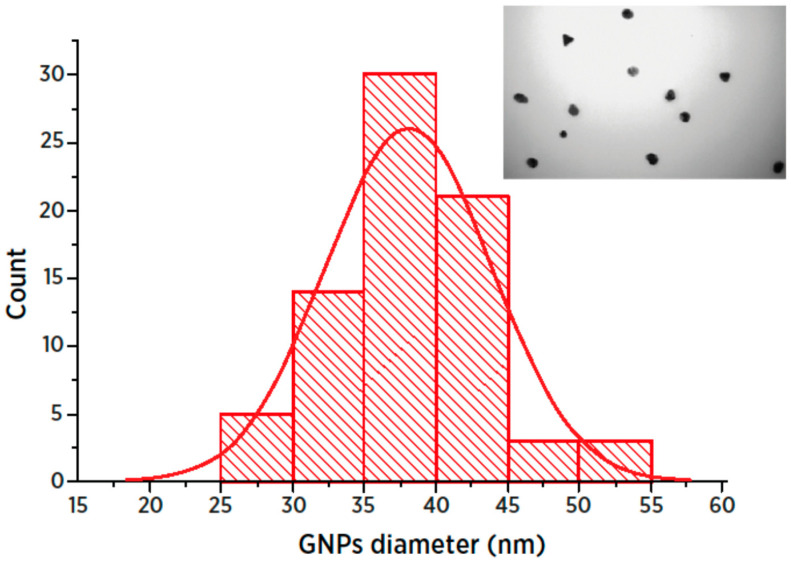
Fragment of gold nanoparticles (GNP) microphotograph and histogram of particle size distribution.

In the course of the LFIA’s development, GNP preparations were obtained. Regarding their characteristics, using transmission electron microscopy (Figure 1), the average diameter for 76 particles was found to be 38.1 ± 5.8 nm (minimum 25.4 nm; maximum 54.1 nm). The particles were not aggregated and were suitable for bioanalytical use.

Monoclonal antibodies IS7 and 6F9 were first characterized using the sandwich ELISA. The chosen optimal regime of the assay (see Section 2.3) provides the detection of bovine TnI in concentrations up to 6 ng/mL (Supplementary Materials, Figure S1). This high sensitivity of TnI determination in ELISA indicated that the antibodies were suitable for the development of the LFIA. The antibodies IS7 and 6F9 were applied in our previous study [17] to control TnI as a biomarker of meat sources by the ELISA technique. The published [17] data confirm that the antibodies interact with troponins of cow, pig, sheep, horse, did not demonstrate binding for the cases of chicken, turkey and ducks. These results allow us to use the same set of antibodies in a rapid test (LFIA) for distinguishing meat sources of mammalian and bird origin.

**Figure 2:**
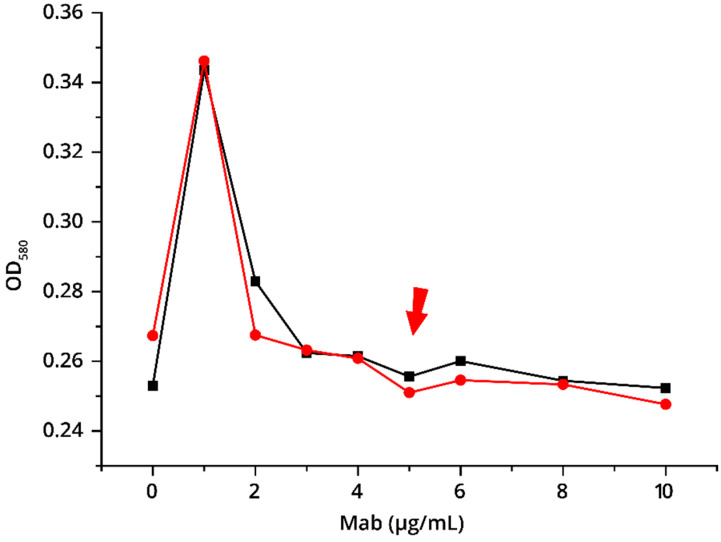
Choice of the concentration of specific antibodies used for conjugation with GNPs (OD_520_ = 1.0): black line—antibody IS7, red line—antibody 6F9. The selected antibody concentration (marked by the arrow) was 5 μg/mL.

Conjugates of GNPs with specific antibodies to troponin I IS7 and 6F9 were obtained. The conditions for the conjugation of antibodies to GNPs were selected based on photometric data after the addition of 10% NaCl. The registered OD_580_ values in these experiments accord wth the aggregated (blue) state of GNPs. In accordance with Hermanson’s [20] recommendations, OD_580_ is considered as a parameter reflecting the surface of GNPs being uncovered by immobilized proteins, and therefore unstable after salt (10% NaCl) addition. For conjugation, a concentration of antibodies was chosen that was 10–20% higher than the OD_580_ exit point on the plateau, which allowed for stabilizing the surface of the GNPs with antibodies [20]. Thus, antibodies were used in the synthesis at a concentration of 5 μg/mL (Figure 2). This is sufficient for a complete monolayer coating of GNPs with antibodies [21]. Excess unreacted antibodies were removed at the stage of the conjugate centrifugation.

### 3.2. Development of the Skeletal TnI LFIA

**Figure 3:**
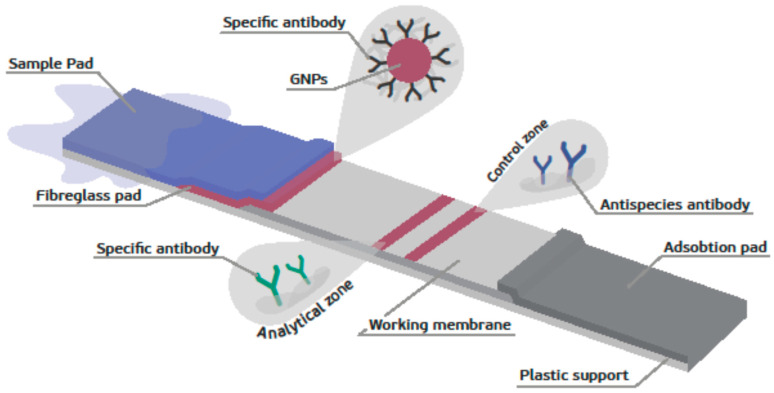
Scheme of the sandwich format LFIA.

A sandwich format was used for the TnI LFIA (Figure 3). This immunoassay format is suitable for assessing native protein and is used for antigens that have at least two epitopes [22].

The development of the assay included optimization of the test system composition and concentrations of immunoreagents used, as well as choosing membrane compounds and regimes for their pretreatment and drying.

**Figure 4:**
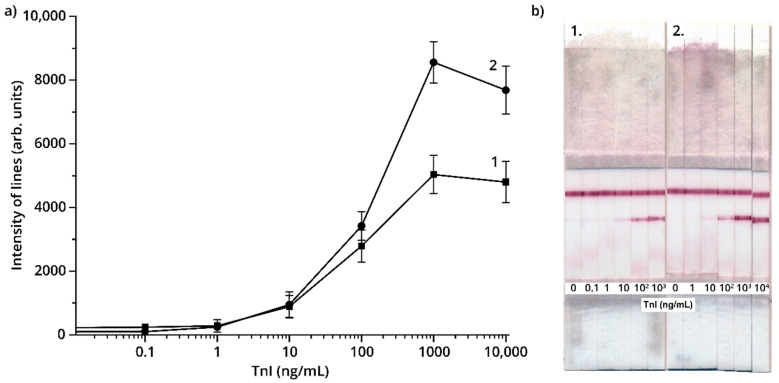
(**a**) Analytical zone intensity (arb. units) vs. TnI concentration using two LFIA variants; (**b**) appearance of the test strips: Variant No. 1 (1); Variant No. 2 (2).

Two orders of antibody incorporation into detectable complexes were considered. In Variant No. 1, antibody IS7 was immobilized on a nitrocellulose membrane, and antibody 6F9 was conjugated to GNPs. In Variant No. 2, immobilized antibody 6F9 and the antibody IS7–GNP conjugate were used. Figure 4 presents the results of the determination of TnI in PBST using these two variants. As can be seen, Variant No. 2 provides a higher intensity of staining of the analytical zones for the same TnI concentrations. Therefore, this order of antibody incorporation into detectable complexes was used for further work.

The LFIA’s sandwich format allows for the use of high concentrations of immunoreagents as a tool for higher sensitivity, so the goal of optimization was to select the concentrations of immobilized antibody and the antibody–GNP conjugate that would maximize signal intensity but would not lead to non-specific coloration outside the binding zones and excessive consumption of reagents.

The LFIA, like ELISA, was performed in PBST, to which 0.5 M KCl was added [17] because, as we have demonstrated for LFIA, this produced a twofold increase in analytical signal intensity without a background signal.

It was shown that the optimal staining intensity of the test zone was obtained by applying 2.5 mg/mL of antibody 6F9 in 10 mM Tris-HCl, pH 9.0, to the membrane. A further increase in antibody concentration did not cause reliable changes. Rabbit antibodies against mouse IgG (0.5 mg/mL in PBS) were used to form the control zone.

As a working membrane, nitrocellulose membranes CNPC-SS12 (Advanced Microdevices), HF090 (Millipore), and HF120 (Millipore) were tested. The CNPC-SS12 membrane was used because it facilitated more even staining of the test zones.

**Figure 5:**
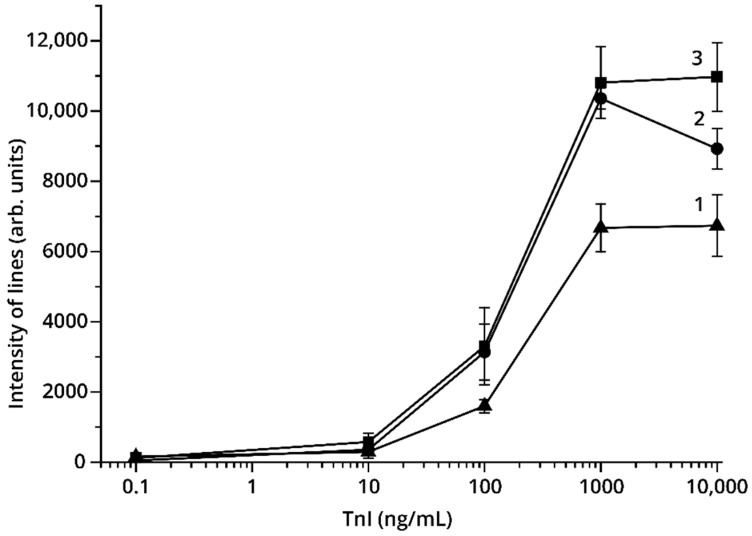
LFIA of TnI: concentration dependences for analytical zone intensity (arb. units) using different fiberglass membranes: 1—Millipore GFDx203000; 2—Ahlstrom-Munksjö 8951; 3—Advanced Microdevices PT-R7 (Variant No. 2 of the test system).

The antibody IS7–GNP conjugate was applied to Advanced Microdevices PT-R7 (application load 16 μL/cm), Millipore GFDx203000 (application load 32 μL/cm), and Ahlstrom-Munksjö 8951 (application load 27 μL/cm) fiberglass membranes at a concentration that corresponded to OD_520_ = 6.0 for the PT-R7 membrane and OD_520_ = 3.0 for the remaining membranes. When using the GFDx203000 membrane, the staining intensity of the analytical zone was minimal as compared with other tested membranes for the same TnI concentrations (Figure 5). With the 8951 membrane, the most uniform distribution of the conjugate and uniform coloring of the binding zone was obtained, therefore that membrane was chosen for test-strip production.

As a membrane for the sample, the GFB-R4 membrane and the Millipore GFDx203000 fiberglass membrane were tested.

In a comparison of drying temperatures for the nitrocellulose membrane with deposited immunoreagents, drying at 37 °C, versus room temperature, showed a 40% increase in analytical zone staining.

**Figure 6:**
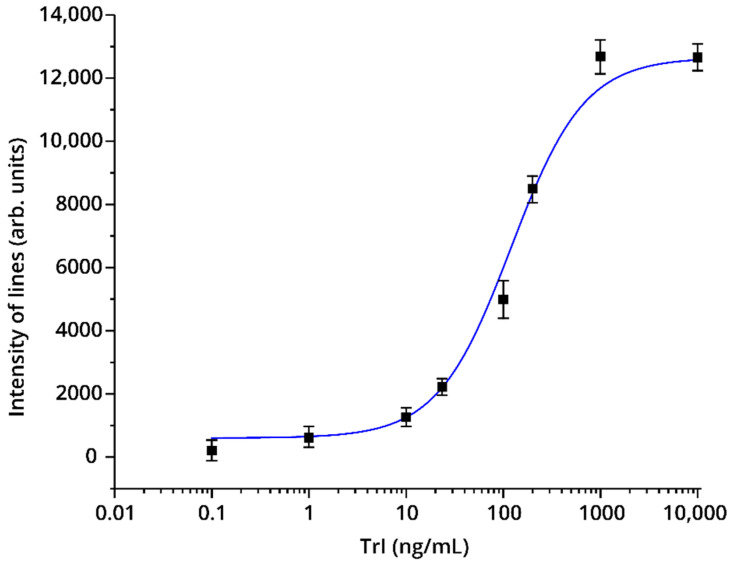
Calibration curve for determining bovine skeletal TnI by LFIA in PBST containing 0.5 M KCl (Variant No. 2 of the test system).

Figure 6 shows the calibration curve of the TnI LFIA under the optimized conditions (see Section 2.6). The assay is characterized by a TnI detection limit of 25 ng/mL.

### 3.3. Analysis of Meat Extracts

**Figure 7:**
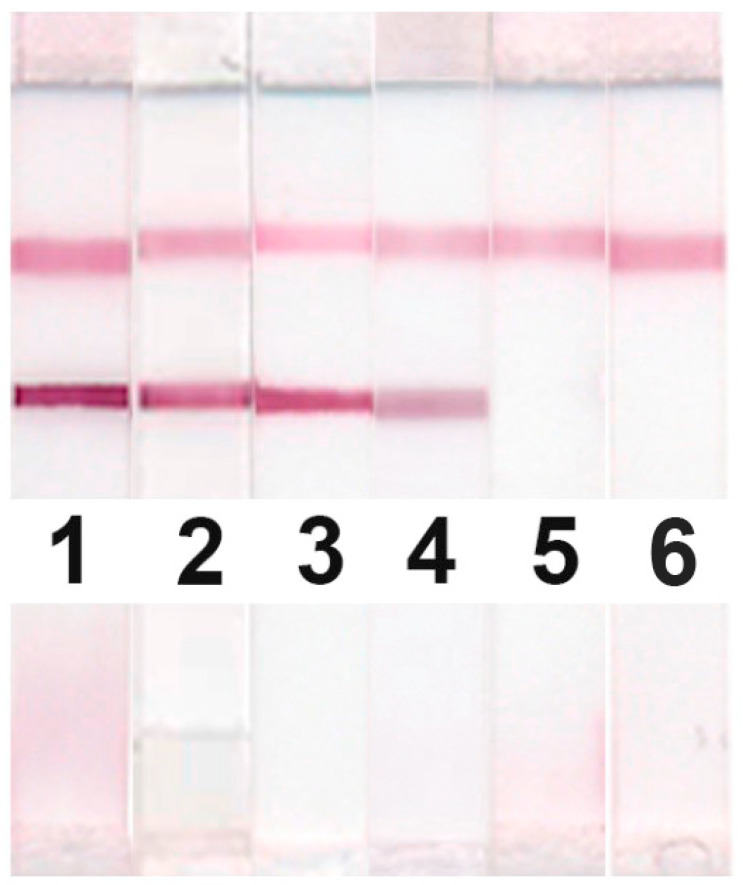
Testing mammalian (1—beef, 2—pork, 3—lamb, 4—horse) and bird (5—chicken, 6—turkey) ten-fold diluted meat extracts by the developed LFIA of TnI (Variant No. 2 of the test system).

Based on the optimization results, test strips were prepared, and a determination of TnI in standard solutions and extracts obtained from meat of various mammal species (beef, pork, lamb, horse) and birds (chicken, turkey) was implemented. The extraction technique previously proposed by the authors for the TnI ELISA was used [17]. The high sensitivity of the TnI LFIA allows for the use of significant (up to 1000 times) dilutions of the testing extracts. It was shown that the developed LFIA recognizes mammalian meat TnI (beef, pork, lamb, horse) but does not detect TnI in poultry meat (chicken, turkey) (See Figure 7). All ten mammalian meat extracts (preparations from beef—4 samples, pork—3 samples, lamb—2 samples, and horse—1 sample) demonstrate the close intensities of test lines for the same dilution. Thus, the deviation from the average level for dilution 1:10 did not exceed 10%.

**Figure 8:**
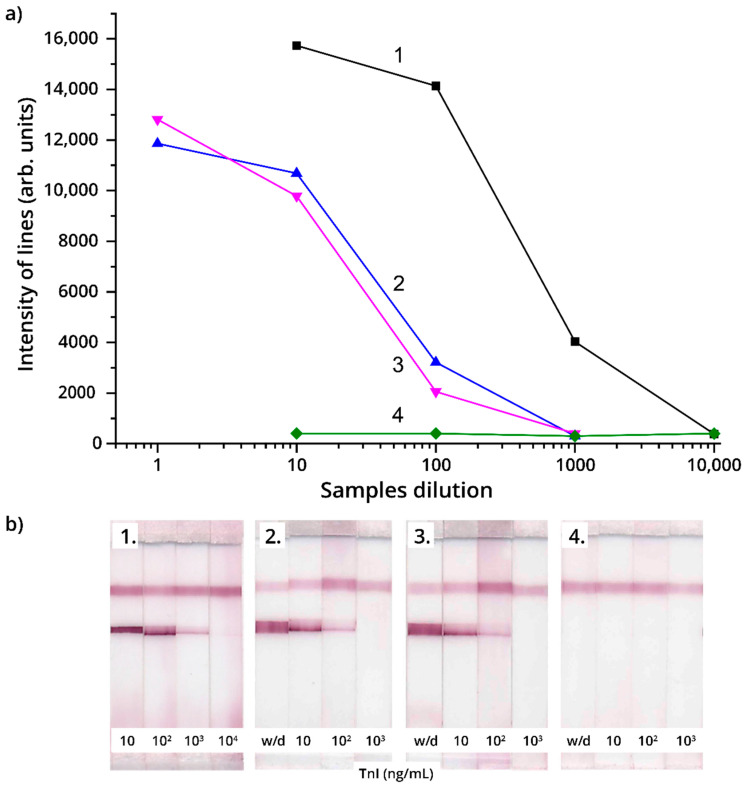
(**a**) Relationship between analytical zone color intensity (arb. units) and meat product sample dilution; (**b**) appearance of the test strips: 1—beef extract (Variant No. 2 of the test system), 2, 3—extracts from sausage samples (Variant No. 2 of the test system), 4—chicken meat extract (Variant No. 2 of the test system).

The dependences on the sample dilution obtained for different mammalian species were quite similar. Figure 8 confirms strong distinguishing mammalian and bird meat (items 1, 4 at a and b in the Figure) and applicability of the developed LFIA to detect mammalian meat in the finished products (sausages) in accordance with their stated composition (items 2, 3 at a and b in the figure).

### 3.4. Analysis of Model Meat Mixes

**Figure 9:**
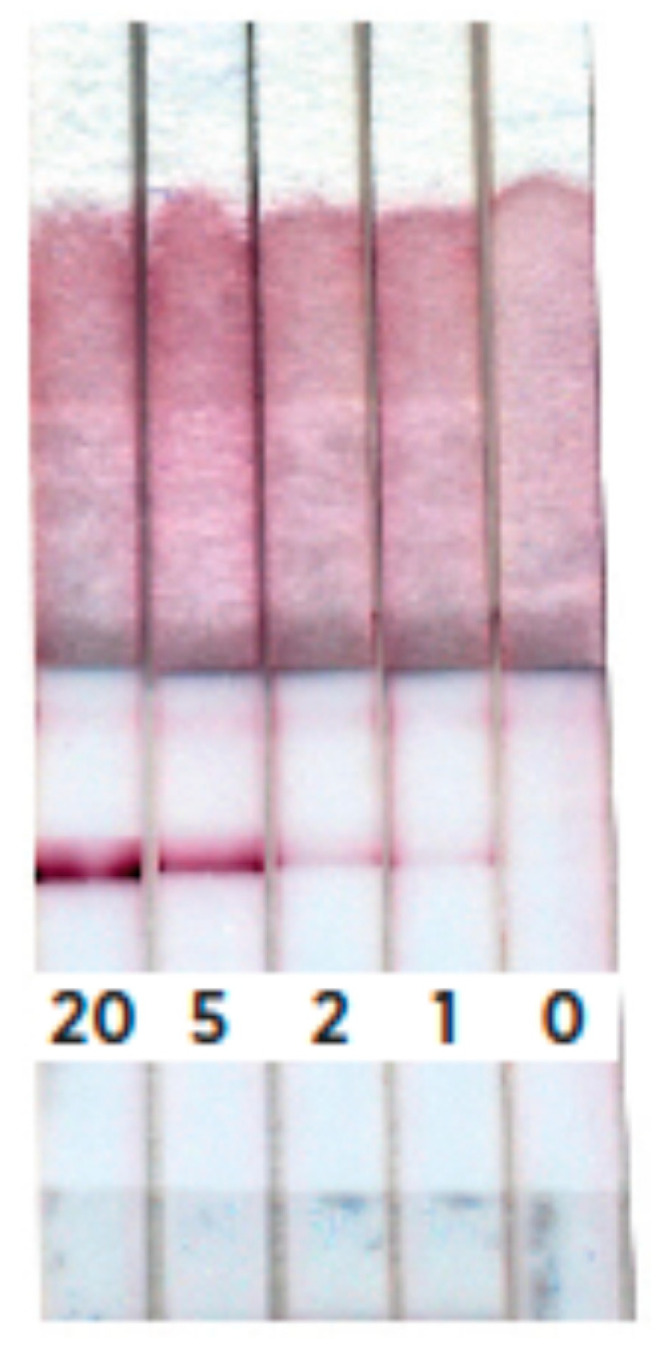
Appearance of the test strips for samples containing 20%, 5%, 2%, and 1% of minced beef meat added to minced chicken meat (*n* = 3).

The developed protocol was used to characterize extracts of model meat mixtures in which 20%, 5%, 2%, 1% and 0.5% of ground beef were added to minced chicken meat (Figure 9). This showed that reliable staining of the analytical zone appears when testing the extract of minced chicken containing 1% beef or more. Good reproducibility of the results of photometric detection was demonstrated. The relative standard deviation of the measured intensity of the analytical lines for all tested samples was less than 14.7%.

### 3.5. Development of Diluting Buffer for Analysis of Sausages

During the analysis of the sausages, a vegetarian sausage (“wheat sausage”), which does not contain raw meat, was used as a control. This showed that the calibration curves for the determination of TnI obtained in the buffer (PBST + 0.5 M KCl) and in the vegetarian sausage extract differed (see the corresponding comparison for samples containing 100 ng/mL of TnI in Table 1, lines 1 and 2). Coloration in the buffer was two times lower than in the vegetarian sausage extract for the same concentrations of TnI. This difference inevitably leads to a distortion of the results of the quantitative determination of TnI.

To reduce the matrix influence, when conducting the LFIA, BSA and the main component used in the production of sausages were tested as additives for the diluting buffer (PBST + 0.5 M KCl). It was found that BSA caused an additional decrease in the analytical signal and even more distortion in the assay results (line 3 of Table 1.) The main components included in the sausage recipes that could affect the LFIA results—sodium chloride, sodium nitrite, and sucrose—were also characterized. It was shown that the addition of sodium nitrite (0.015–0.04 M) or sucrose (0.003–0.01 M) to the base buffer for analysis (PBST + 0.5 M KCl) increases the staining intensity of the analytical zones slightly, while the combined use of 0.04 M sodium nitrite and 0.01 M sucrose gives an increase in intensity of 10%. The addition of NaCl to the buffer did not affect the staining, which can be explained by the high concentration of chloride ions in the initial buffer. This showed that the use of a buffer containing 0.04 M NaNO_2_ and 0.01 M sucrose does not reach the signal value for undiluted vegetarian sausage extract (see lines 4 and 1 of Table 1), but the math of the results can be used for comparison of the assay data, with the calibration curve obtained in extracts of vegetarian sausage when diluted 50 and 75 times (lines 5–7 of Table 1 present the corresponding comparison of different dilutions of the extract). Thus, the use of the calibration curve obtained in the complex buffer (PBST containing 0.5 M KCl, 0.04 M NaNO_2_, 0.01 M sucrose) in the analysis of meat product extracts diluted 50 times or more provides the absence of the distorting influence of the sample matrix and the possibility of correct determination of the TnI content in meat products.

### 3.6. Sausages Analysis

The developed protocol was used to analyze the different types of sausage. The sausages were prepared for testing in accordance with the Russian state standard R 55455-2013—boiled-smoked meat sausages, commonly defined specifications used in recipes of meat products. Based on these formulations, the main components (such as sodium chloride, sodium nitrite, sucrose, ground black and white pepper, ground cardamom, ground nutmeg) and their characteristic concentrations were selected. The use of such a universal matrix allowed us to verify the suitability of the developed test when working with products containing typical additives used in industrial technologies.

The resulting optical densities for diluting a sample of meat products are presented in Figure 7 (curves 3, 4). Table 2 presents the results of calculating the amount of TnI basing on the recipe composition and on the LFIA results. It was shown in [17] that 1 g of pork contains 0.7 mg of TnI, and 1 g of beef contains 0.46 mg of TnI. This made it possible to transform the amounts of meat raw materials used in the production of sausages to TnI levels.

The TnI determination in sausage extracts by the LFIA was performed by diluting the extracts and recalculating using the TnI calibration curve (for PBST containing 0.5 M KCl, 0.04 M NaNO_2_, and 0.01 M sucrose). As can be seen from Table 2, the developed LFIA reveals 82–93% TnI. Thus, the developed assay is able to control 20% deviation from the declared composition of meat products.

## 4. Conclusions

An immunochromatographic technique in a sandwich format using gold nanoparticles as labels for determining skeletal troponin I levels was developed. This technique is characterized by a detection limit of 25 ng/mL, and it takes only 15 min. The test system is specific to the mammalian TnI (beef, pork, lamb, horse) and does not detect avian TnI (chicken, turkey), thus allowing differentiation of meat from mammals and birds. The developed technique can detect 1% of minced beef in minced chicken. The feasibility of determining TnI in finished meat products that have passed through technological processing (smoking–cooking–smoking, cooking and smoking) is shown. The TnI determination in sausage extracts was carried out using a PBST buffer containing 0.5 M KCl, 0.04 M NaNO_2_, and 0.01 M sucrose. The use of this diluting buffer eliminates the influence of the sample matrix on the measurement results. The developed test system can assess the content of mammalian muscle tissue in minced heat-treated meat products and compare the obtained values with the claimed composition. The developed LFIA allows for the reveal of 82–93% TnI with respect to the recipe composition of meat mixtures. The developed technique seems to be very promising for controlling the conformity of meat products’ composition.

## Figures and Tables

**Table 1:** Staining intensities of the test strip analytical zone at TnI concentration of 100 ng/mL in various matrices and diluting solutions (%); *n* = 3.

	Matrices	Intensities, % *
1	Vegetarian sausage extract	60 ± 8
2	PBST containing 0.5 M KCl	32 ± 6
3	PBST containing 0.5 M KCl and BSA	10 ± 4
4	PBST containing 0.5 M KCl, 0.04 M NaNO_2_, 0.01 M sucrose	40 ± 6
5	Extract of vegetarian sausage diluted 20 times	58 ± 8
6	Extract of vegetarian sausage diluted 50 times	42 ± 5
7	Extract of vegetarian sausage diluted 75 times	39 ± 7

* The value of 100% accords to upper plateau of the calibration curve reached for 1000 ng/mL of TnI in PBST containing 0.5 M KCl, 0.04 M NaNO_2_, 0.01 M sucrose.

**Table 2:** Amounts of TnI, calculated from the added raw materials and from the LFIA data.

Composition of Sausages	According to the Recipe Composition, mg/g	According to the LFIA Results	
		mg/g	% of Bookmark
Sample 1 (smoked—cooked—smoked sausage), 0.75 g of raw meat (beef) per g of sausage	0.35	0.290 ± 0.018	82
Sample 2 (cooked and smoked), 1 g of raw meat (0.3 g of beef and 0.7 g of pork) per g of sausage	0.62	0.582 ± 0.004	93

## Supplementary Materials

The following are available online at https://www.mdpi.com/2304-8158/9/11/1662/s1, Figure S1. Calibration curve of the bovine TnI detection by sandwich ELISA. Table S1. Composition of the smoked–cooked–smoked sausage, Sample 1. Table S2. Composition of the cooked and smoked sausage, Sample 2.
