# Supplementary material for: Lateral Flow Immunoassay to Detect the Addition of Beef, Pork, Lamb, and Horse Muscles in Raw Meat Mixtures and Finished Meat Products
Source: Foods. 2020 Nov 13;9(11):1662. doi: 10.3390/foods9111662 (PMC7697940; doi:10.3390/foods9111662)
Supplement: Supplementary file 1 [file foods-09-01662-s001.pdf]

Supplementary Materials

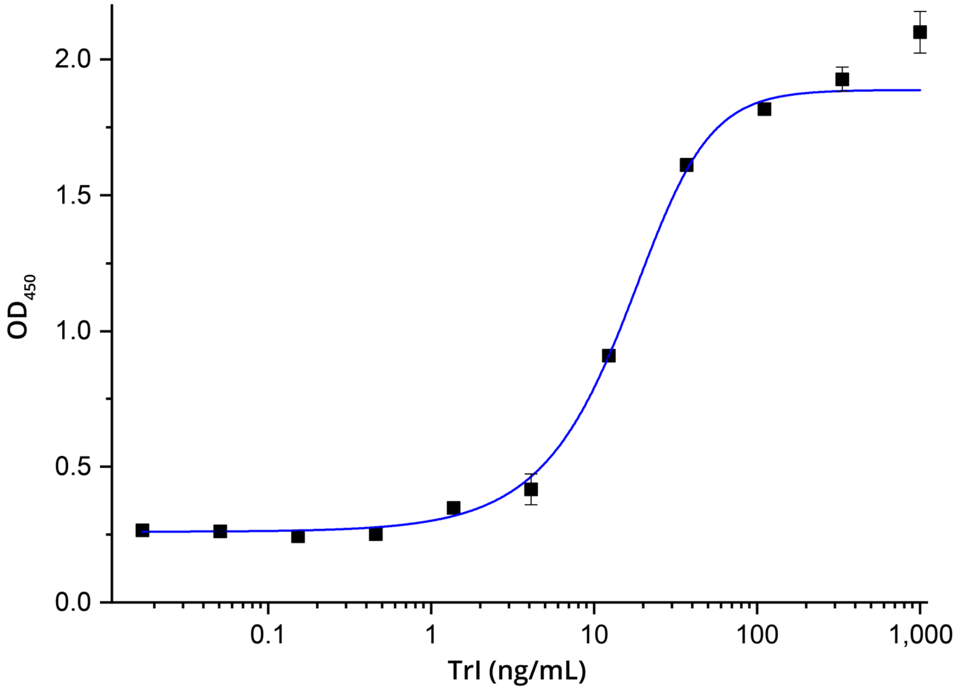

Figure 1. Calibration curve of the bovine TnI detection by sandwich ELISA. The measurements were made in triplicate.

Table 1. Composition of the smoked – cooked – smoked sausage, Sample 1.

	Ingredients	g/kg
1	Top grade trimmed beef	750.00
2	Pork back fat, pieces no more than 6 mm	250.00
3	Sodium chloride	30.00
4	Sodium nitrite	0.10
5	Granulated sugar	2.00
6	Ground black or white pepper	1.50
7	Cardamom or ground nutmeg	0.30

Table 2. Composition of the cooked and smoked sausage, Sample 2.

	Ingredients	g/kg
1	Beef	300.00
2	Pork semi-fat	400.00
3	Bacon	30.00
4	Sodium chloride	10.00
5	Sodium nitrite	10.00
6	Sugar	1.00
7	Ground black pepper	0.60
8	Ground allspice	0.50
9	Garlic	0.65
